# Quantitative Study on the Adsorption State of n-Octane in Kaolinite Slit-like Pores Based on Four Angular Parameters

**DOI:** 10.3390/molecules30183739

**Published:** 2025-09-15

**Authors:** Fang Zeng, Shansi Tian, Zhentao Dong, Hongli Dong, Bo Liu, Valentina Erastova, Haiyang Liu

**Affiliations:** 1 The State Key Laboratory of Continental Shale Oil, Northeast Petroleum University, Daqing 163318, China; fang.zeng@nepu.edu.cn (F.Z.); b20010024@s.upc.edu.cn (Z.D.); hongli.dong@nepu.edu.cn (H.D.); liubo@nepu.edu.cn (B.L.); 2 The Artificial Intelligence Energy Research Institute, Northeast Petroleum University, Daqing 163318, China; 3 The Heilongjiang Provincial Key Laboratory of Networking and Intelligent Control, Northeast Petroleum University, Daqing 163318, China; 4 Institute of Unconventional Oil & Gas, Northeast Petroleum University, Daqing 163318, China; 5 School of Chemistry, University of Edinburgh, David Brewster Road, Edinburgh EH9 3FJ, UK; valentina.erastova@ed.ac.uk; 6 School of Geosciences, China University of Petroleum (East China), Qingdao 266580, China; bz24010008@s.upc.edu.cn

**Keywords:** n-octane, kaolinite, slit-like pores, adsorption configuration, angular parameters, molecular simulation

## Abstract

Shale oil extraction efficiency hinges on the interfacial interactions between oil molecules and reservoir clay minerals, such as kaolinite, whose slit-like pores serve as primary storage spaces for alkanes. This study introduces a novel multi-dimensional quantification method using four angular parameters—elevation angle (θ), azimuth angle (φ), rotation angle (ω), and dihedral angle (τ)—to systematically investigate the adsorption configuration of n-octane in kaolinite slit pores ranging from 0.45 to 14.05 nm. Through molecular simulations and advanced trajectory analysis, we elucidate the impact of pore sizes on alkane adsorption density, layering, and molecular configurations. Results reveal that pore size regulates molecular behavior via steric hindrance and potential field superposition, while the four angular parameters can effectively capture subtle changes in. this molecular behavior: (1) the elevation angle (θ) around 0° indicates complete alignment parallel to surface, but is modulated at increasing distance from the surface into the pore-region highlighting a disordered state; (2) the azimuth angle (φ) is concentrated at 60° and 120° on the siloxane tetrahedral surface due to lattice regulation, but shows a disordered distribution on the hydroxyl octahedral surface; (3) the rotation angle (ω) is mainly concentrated at 0° and 90° indicating molecular plane being either parallel or perpendicular to the surface; (4) the dihedral angle (τ) remains at ~0°, indicating that the molecular chains are straight. In pores smaller than 4.26 nm, strong confinement yields ordered molecular arrangements (θ = 0°, φ at 60° or 120°, ω = 0°) with high adsorption density; for larger pores than 4.26 nm, disordered configurations and increased layering (up to eight layers) with stable density and adsorption capacity per unit area are observed. The proposed parameter system overcomes limitations of traditional qualitative approaches, offering a standardized, scalable tool for quantifying alkane-clay interactions. This framework enhances understanding of shale oil occurrence mechanisms and supports optimized extraction strategies, with broad applicability to other chain molecules and 2D materials in interface science.

## 1. Introduction

Shale oil, as a globally important unconventional energy resource, exhibits occurrence and extraction efficiency behaviors governed by molecular interactions at the interface between oil components and reservoir clay minerals [1]. Kaolinite is one of the most abundant clay minerals in shale and serves as the main storage space for shale oil (comprised mainly of alkanes) [1]. Kaolinite (Al_2_Si_2_O_5_(OH)_4_) features a layered structure with slit-like pores formed by the alternating arrangement of siloxane tetrahedra and hydroxide octahedra. The adsorption of alkanes within these pores is influenced by pore size, surface chemistry, and molecular configurations, orientation, conformation, and ordering, which determine adsorption strength, diffusion dynamics, and recovery efficiency [2,3].

While prior studies have explored macroscopic adsorption capacity, isotherms, and thermodynamic parameters, or qualitatively described molecular arrangements such as “lying flat” or “vertical alignment” via molecular simulations [4,5], these approaches lack the resolution to capture molecular-scale configurational details or quantify the impact of pore size variations. However, key questions remain: How does alkane orientation evolve across nano- to micrometer-scale kaolinite pores? How does the surface lattice, such as the six-membered siloxane rings, influence molecular planar arrangement? Does molecular chain torsion vary with pore confinement? To address these, we propose a multidimensional quantification framework based on four angular parameters: elevation angle (θ), azimuth angle (φ), rotation angle (ω), and dihedral angle (τ).

Prior interfacial-orientation analyses typically rely on a single tilt angle relative to the surface normal and its Legendre order parameter P_2_ or on a second-moment orientation tensor to summarize alignment [6,7]. While valuable, such averages can map distinct angular distributions to the same scalar value, obscuring registry and symmetry-specific features at interfaces. Two-angle schemes (tilt + azimuth), widely used in surface spectroscopy, improve matters but still lack sensitivity to molecular plane–surface rotation and backbone dihedral flexibility, which are central to packing and layering in confinement [8,9]. For kaolinite specifically, the (001) siloxane basal surface exhibits a hexagonal lattice of six-membered rings [10], making in-plane lattice commensurability a first-order effect that single-parameter metrics cannot capture. By contrast, our four-angle framework—elevation θ (out-of-plane tilt), azimuth φ (in-plane registry to a lattice vector), plane rotation **ω** (molecular plane vs. surface), and dihedral τ (trans–gauche content)—decouples surface-normal alignment, lattice commensurability, plane alignment, and chain flexibility. This is particularly pertinent for the kaolinite siloxane (001) face, whose hexagonal Si–O ring symmetry can enforce preferred in-plane orientations, a feature that single-parameter metrics cannot capture.

We demonstrate the use of the method on the n-octane system, as an example of a typical short-chain alkane in shale oil, within kaolinite slit-like pores of widths between 0.45 and 14.05 nm. This molecular dynamics study aims to (1) elucidate the effects of pore size on n-octane adsorption density and layering, (2) quantify the molecular orientation and conformational responses of n-alkane to pore size via the four angular parameters, and (3) establish a robust, transferable method for analyzing alkane-clay interactions, advancing the understanding of shale oil occurrence and optimizing extraction strategies.

## 2. Materials and Methods

### 2.1. System Setup

**Figure 1:**
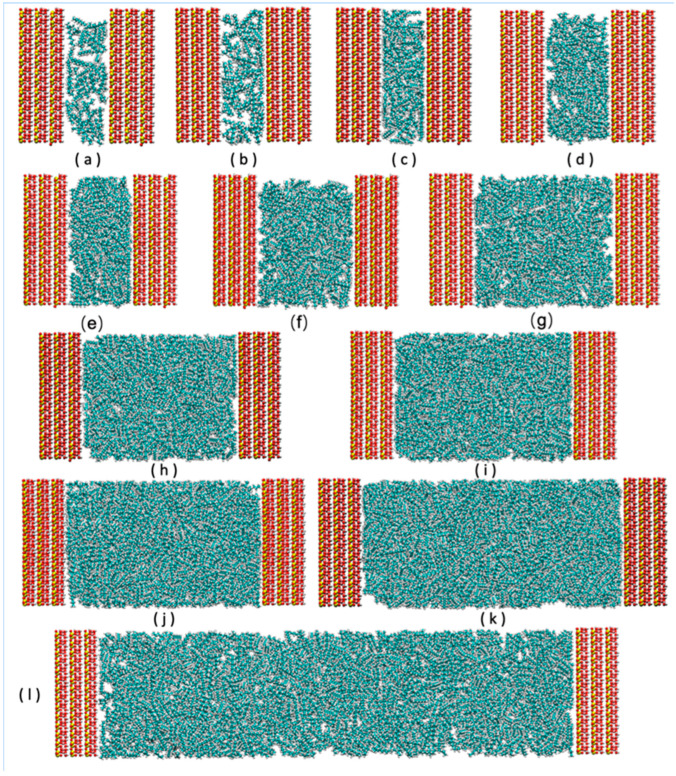
Initial setup of a model of adsorption of n-octane with different molecular numbers in kaolinite slit pores. (**a**) 70; (**b**) 80; (**c**) 140; (**d**) 200; (**e**) 275; (**f**) 400; (**g**) 600; (**h**) 800; (**i**) 1000; (**j**) 1200; (**k**) 1600; and (**l**) 2000 n-octane molecules. Colors: n-octane—C (cyan) and H (white); kaolinite framework—O (red), Si (yellow), Al (pink).

Kaolinite clay (KAO) with the stoichiometry Al_2_Si_2_O_5_(OH)_4_ was retrieved from the American Mineralogist Crystal Structure Database (AMCSD) [11]. The KAO surface was constructed as a grid of 12 × 7 unit cells, each of 5.1540 × 8.9420 Å^2^, resulting in an area of 62 × 63 Å^2^. The individual layer thickness of KAO is 6.3910 Å, and a stack of 3 layers was used in this study, giving a total clay thickness of 19 Å. The clay layer was aligned on the *xy*-plane, with the interlayer space extending along the z-axis. Different numbers of n-octane molecules (50, 70, 80, 100, 120, 140, 160, 180, 200, 225, 250, 275, 300, 325, 350, 375, 400, 600, 800, 1000, 1200, 1600, and 2000) were intercalated into the interlayer space, and the system relaxed under the NPT ensemble (323 K, 100 bar, detailed below), leading to slit-like pores of varying d-spacings (Figure 1).

Pore-width selection and geological representativeness. The targeted slit widths (0.45–14.05 nm) span sub-nanometer tactoid/interlayer gaps up to mesoporous interparticle slits commonly reported for kaolinite aggregates in lacustrine shales. Sub-1 nm pores capture strong confinement regimes; widths ≥ 4 nm represent multi-layer adsorption with diminished wall–wall potential overlap.

Initialization to mitigate packing bias in ultra-narrow slits. For each width, we generated three independent replicas with different random seeds. Sub-1 nm systems were pre-packed by low-load insertion followed by steepest-descent minimization and short NVT compression (0.5–1 ns) before NPT relaxation, which reduces initial overlapping and bias. All simulations employ 3D PBC with a semi-isotropic cell (x–y coupled, z independent).

The pore walls expose an ideal siloxane (001) basal face of kaolinite under periodic boundary conditions and do not include steps, edges, or point defects/impurities. This choice isolates lattice-registry effects (hexagonal Si–O rings) and provides a best-case upper bound for in-plane ordering. Natural kaolinite surfaces are known to display steps and edge terminations and to exhibit spatially varying charge arising from edge deprotonation and occasional isomorphic substitutions, which alter local electrostatics and corrugation of the surface potential. Such heterogeneity is expected to broaden the azimuthal distribution (φ) and increase the dispersion of plane rotation (ω) relative to our ideal model. We therefore interpret the present φ–ω ordering as an upper limit and discuss anticipated deviations for heterogeneous surfaces in the Section 4.

### 2.2. Force Field Parameters

The ClayFF force field [12], specifically developed for modeling clay-like minerals, was employed to model KAO. For alkanes, the CHARMM36 force field [13] was used, with force field parameters assigned via the CGenFF algorithm [14]. Cross interactions between ClayFF (kaolinite) and CHARMM36 (n-octane) were treated via Lorentz–Berthelot combining rules for Lennard-Jones parameters with full PME electrostatics. Bond constraints involving H followed the respective force-field defaults (LINCS/SHAKE). The 1–4 scaling factors were used for the native settings of CHARMM36; ClayFF partial charges and hydroxyl flexibility were preserved. This combination of force fields has been previously validated and applied by the authors [1,15,16,17].

### 2.3. Simulations

All simulations were performed using the GROMACS 4.6.7 software package (GROMACS Development Team, Uppsala University, Uppsala, Sweden) [18]. Steepest-descent minimization was followed by staged equilibration and production as below. Velocity–Verlet with a 1.0 fs timestep; bonds to H constrained (LINCS). PME electrostatics (FFT grid spacing ~0.12 nm, 4th-order interpolation); Lennard–Jones cutoff 1.2 nm with a switching function from 1.0 to 1.2 nm; long-range dispersion correction applied to energy and pressure.

We used Berendsen coupling only for pre-equilibration and a Nosé–Hoover thermostat (τT = 1.0 ps) with a semi-isotropic Parrinello–Rahman barostat (τP = 5.0 ps; compressibility 4.5 × 10^−5^ bar^−1^) for production, which yields correct NPT statistics. Unless otherwise noted, all reported structural and orientational properties were averaged from production trajectories at 323 K and 1 bar. The brief pre-compression at 100 bar was used only to set the target d-spacing, after which the system was fully relaxed at 1 bar before any averaging. The present study, therefore, targets ambient-to-moderate laboratory conditions that are commonly used as a baseline for clay–hydrocarbon interfaces. For two representative widths (≈0.9 nm and ≈5 nm), we performed short scans at 298/323/373 K and 1/500 bar (≥10 ns each) to test robustness; procedures are identical to the main protocol.

**Figure 2:**
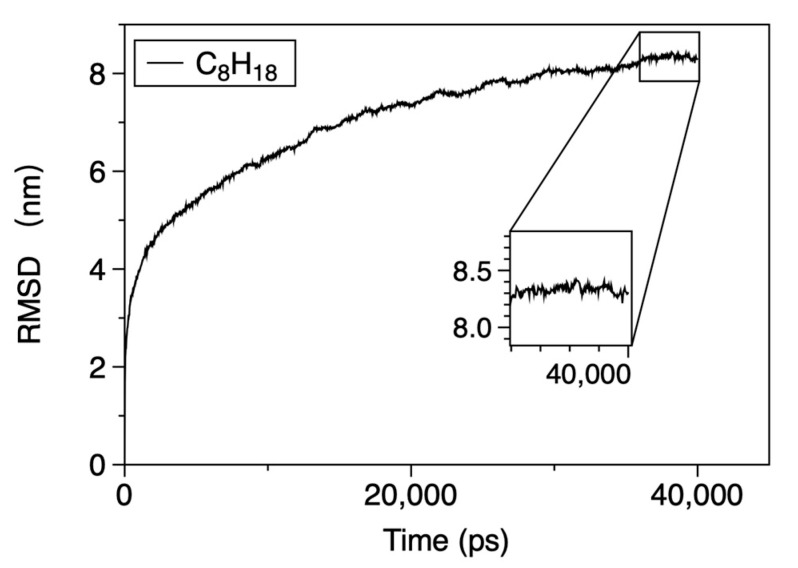
Structural RMSD convergence. Time evolution of the RMSD of n-octane (2000 n-octane molecules). The curve asymptotes at 40 ns, which we take as the operational convergence point for subsequent averaging.

Sampling length, replicas, and convergence. For each width, three replicas (independent initial velocities/seeds) were run for ≥40 ns in production. We monitored convergence using the root-mean-square deviation (RMSD) of n-octane heavy atoms after aligning each frame to the kaolinite lattice to remove rigid-body motion. The RMSD exhibits a clear plateau at 40 ns, beyond which only small fluctuations remain (Figure 2). Unless otherwise noted, all averages are computed after this RMSD plateau.

### 2.4. Analysis

Basal interlayer d-spacing and partial density analyses were carried out using GROMACS tools. All analyses designed to describe molecular alignment relative to the surface were developed in-house, utilizing TCL scripts in VMD 1.9.2 (University of Illinois at Urbana–Champaign, Urbana, IL, USA) to parse simulation trajectories and Python 2.7 (Python Software Foundation, Beaverton, OR, USA) [19] with numpy (Version 1.21) and matplotlib (Version 2.10) packages [20] for data analysis and plotting. Dihedral angle analysis reports all dihedral angles as a function of distance from the clay surface.

**Figure 3:**
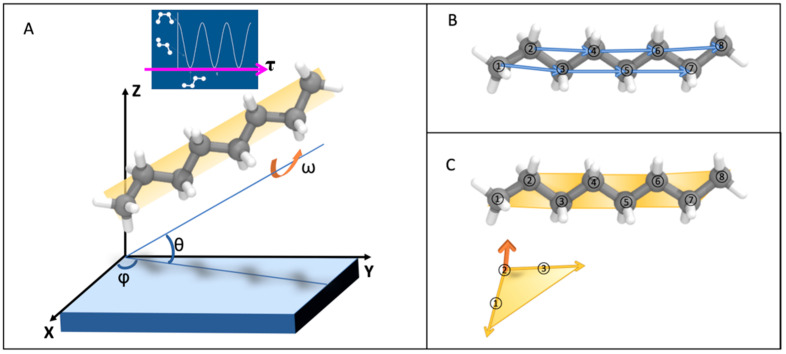
Geometry of the four angles. Elevation θ (axis vs. plane), azimuth ϕ (in-plane registry), plane rotation $\omega =\arccos\left(\left|n_{m}\cdot n_{s}\right|\right)$, and backbone torsion $\tau =\left\langle angdiff\left(\varphi_{i},180^{\circ }\right)\right\rangle$ with trans = $180^{\circ }$, gauche = $\pm 60^{\circ }$. Illustrations of (**A**) four angle parameters used in this paper to define the alignment of n-octane; (**B**) number of carbon atoms in n-octane (from left to right: C1, C2, C3, C4, C5, C6, C7, C8); (**C**) example of n-octane at ω = 0°.

To quantify the alignment of alkanes with respect to the xy plane, we adapted and built upon the orientation–angle analysis introduced in [21] originally for crystallization studies and computed four angular order parameters (Figure 3A).

**Elevation angle (θ):** The angle between the vector (defined within each molecule between two carbon atoms separated by one atom, e.g., C1 and C3, C2 and C4, etc., as shown in Figure 3B) and the *xy*-plane. This parameter characterizes the degree of inclination of the molecular chain relative to the clay surface.

**Azimuth angle (φ).** The angle between the projection of the chain-axis vector u onto the xy plane and the *x* axis, describing the in-plane orientation of the molecular chain relative to the clay crystallographic axes (Figure 3A).

**Rotation angle (ω):** To determine this angle, all planes defined by three consecutive carbon atoms in each molecule (shown as the orange surface in Figure 3A) were first extracted. The normal vector of each plane was computed as the cross product of two vectors connecting the three atoms (e.g., C2 to C1 and C2 to C3, shown in orange in Figure 3C). The elevation of this normal vector relative to the z-axis was then calculated and adjusted by 90 degrees, such that 0 degrees corresponds to planes parallel to the clay layer, and 90 degrees corresponds to planes perpendicular to the clay layer. This parameter reflects the rotational state of the molecular plane relative to the surface. Let $n_{s}$ be the unit normal of the kaolinite basal plane (*z*-axis), and a a lattice vector in the basal plane. For each n-octane molecule, we define the molecular axis u as the first principal component (PCA) of heavy-atom coordinates (validated against the C1 to C8 vector). We compute a best-fit molecular plane by a sliding window over three consecutive carbons and obtain its unit, normal $n_{m}$, and define the following:

(1) $$\omega =arccos(|nm\cdot ns|),\omega \in [0^{\circ },90^{\circ }]$$

**Dihedral angle (τ):** The angle between two adjacent planes formed by four consecutive carbon atoms (e.g., **τ1:** C1-C2-C3 and C2-C3-C4, **τ2:** C2-C3-C4 and C3-C4-C5, …) in the alkane chain. It quantifies the torsional conformation of the molecular backbone (the blue area in Figure 3A). Thus τ ≈ 0° indicates a trans-dominated backbone on average, not an all-trans chain at every bond. For each C–C–C–C dihedral φ_*i*_ along the chain, trans corresponds to φ_*i*_ = 180° and gauche to φ_*i*_ ≈ ±60°. We report a molecule-level torsion metric as the mean deviation from trans Equation (2). Contemporary CHARMM-family parameters reproduce realistic trans/gauche equilibria for n-alkyl chains under ambient conditions, consistent with spectroscopic and MD benchmarks; hence, the near-surface reduction of τ reflects confinement-stabilized trans propensity rather than an artificial freezing of dihedrals by the force field.

(2) $$\tau =\frac{1}{N}\sum_{i}\;min\left(\left|\varphi_{i}-180^{\circ }\right|,360^{\circ }-|\varphi_{i}-180^{\circ }|\right)$$

Density profiles and adsorption. The number-density profile was computed as $\rho \left(z\right)=N\left(z\right)/\left(A\Delta z\right)$, normalized as $\left(\rho^{*}\left(z\right)=\rho \left(z\right)/\rho_{\mathrm{bulk}}\right)$. We report an operational characteristic width *H* marking the onset of multilayer decoupling, defined as the smallest simulated slit width at which the normalized density profile shows ≥3 resolvable peaks with nearly constant spacing and the first-layer peak height no longer increases. Because widths were sampled discretely, *H* equals the corresponding simulated value and should be interpreted as grid-limited (precision bounded by the spacing to adjacent widths), not as a fitted critical point.

### 2.5. Visualization

Histograms of elevation, azimuth, rotation angles, and dihedral angles as a function of distance from the clay were generated with a bin size of 1 degree for angles and 1 Å for distance. Snapshots were generated using VMD 1.9.2 [22], with atoms rendered using the following color scheme: the clay structure contains silicon (yellow), oxygen (red), aluminum (pink), and hydrogen (white) atoms; organic molecules contain carbon (cyan), hydrogen (white), and oxygen (red) atoms. Due to the periodicity of the system, the clay layer visually appears on both sides of the box. Plots were made with VMD 1.9.4 using the Tachyon ray tracer.

## 3. Results

### 3.1. Adsorption Density and Layering Response to Pore Size

**Figure 4:**
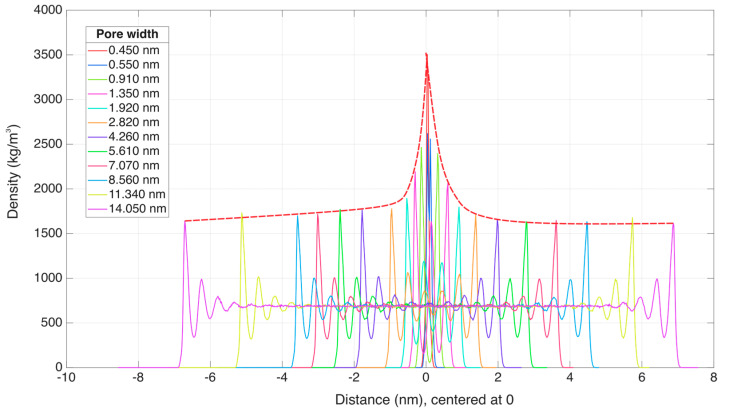
Density curve of n-octane in slit pores of different pore sizes of kaolinite. The red dotted line denotes the envelope curve connecting the maximum adsorption density peaks across different pore sizes.

The adsorption behavior of n-octane in kaolinite slit-like pores strongly depends on pore size, exhibiting distinct density profiles and layering characteristics (Figure 4). Key observations are as follows:

- Peak density variation: In pores < 4.26 nm, peak adsorption density decreases from 3500 kg/m^3^ (0.45 nm) to 1800 kg/m^3^ (4.26 nm) as pore size increases. For pores > 4.26 nm, the peak density stabilizes at ~1800 kg/m^3^. This trend reflects the superposition of van der Waals forces from opposing surfaces in narrow pores, enhancing molecular packing. In wider pores, the absence of such potential field overlap results in a consistent density of peaks, governed solely by single-surface interactions.
- Layering behavior: As the pore size expands from 0.45 nm to 14.05 nm, the number of adsorption layers increases from one to eight. The outer layer exhibits lower peak density than the inner layers due to the diminishing influence of forces, leading to the increased molecular disorder far from the interface.

### 3.2. Quantitative Analysis of n-Octane Configuration Using Angular Parameters

**Figure 5:**
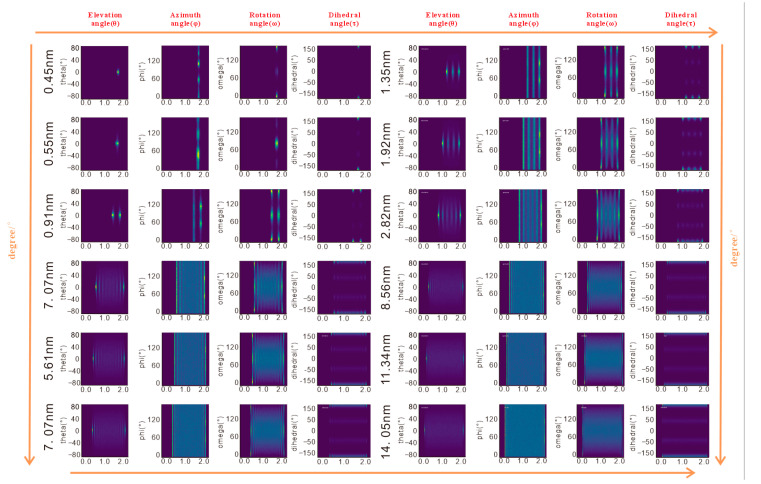
Density profiles and quantitative evaluation of the four angles of n-octane in kaolinite slit pores with different pore sizes. Because the basal faces are ideal and defect-free by construction, the degree of lattice-locked in-plane registry (φ) and low plane-rotation (ω) observed here should be viewed as an upper bound relative to heterogeneous, step-rich natural surfaces.

**Figure 6:**
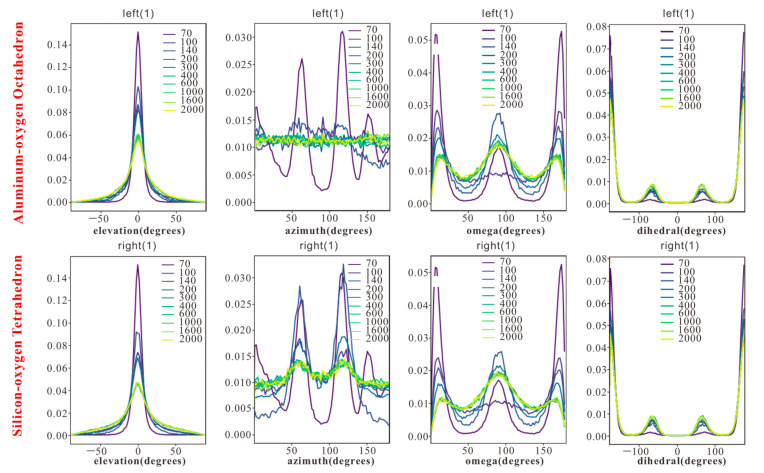
Distribution of angular parameters across aluminol octahedral and siloxane tetrahedral surfaces in kaolinite slit pores.

The four angular parameters—elevation angle (θ), azimuth angle (φ), rotation angle (ω), and dihedral angle (τ)—provide a multi-dimensional framework to quantify configurations of n-octane in response to pore size changes, bridging macroscopic characteristics and microscopic mechanisms (Figure 5 and Figure 6).

#### 3.2.1. Elevation Angle (θ): Molecular Chain Inclination

The elevation angle (θ) quantifies the tilt of the n-octane chains relative to the kaolinite surface, correlating closely to adsorption density:

- Pores < 4.26 nm: θ is concentrated near 0°, indicating near-parallel alignment of the molecular chains with the surface, maximizing contact area and van der Waals interactions, consistent with high-density peaks.
- Pores > 4.26 nm: The first adsorption layer maintains a stable θ distribution ~0° reflecting strong surface adhesion. Outer layers show disorder in θ values, approaching bulk-phase behavior as surface forces weaken, allowing molecules to extend into the pore space.

#### 3.2.2. Azimuth Angle (φ): Molecular Chain Projection on Surface

The azimuth angle (φ) reveals the influence of surface lattice structure on molecular orientation, highlighting significant surface heterogeneity:

- Pores < 0.55 nm: φ concentrates at 60° and 120°, driven by the six-membered ring lattice of siloxane tetrahedra, constraining molecules along its symmetry axes.
- Pores > 0.55 nm: On the siloxane tetrahedral surface, φ remains concentrated at 60° and 120°, while on the aluminol octahedral surface, hydroxyl group vibrations cause a disordered distribution of φ. In the outer layers of larger pores, φ becomes increasingly dispersed due to vanishing lattice forces.

The differential φ distribution underscores the role of surface chemistry in regulating molecular orientation, with a rigid siloxane tetrahedral lattice enforcing order and dynamic hydroxyl groups on the aluminol octahedral surfaces promoting disorder.

#### 3.2.3. Rotation Angle (ω): Molecular Plane Rotation

The rotation angle (ω) reflects the rotational state of n-octane’s molecular plane, primarily governed by steric hindrance (Figure 4):

- Pore size = 0.45 nm: ω is concentrated at 0°, indicating molecular planes align parallel to the surface to minimize steric constraints.
- Pore size = 0.55 nm: ω suddenly changes to 90°, with planes perpendicular to the surface, reducing electrostatic repulsion from the negatively charged aluminol octahedral surface.
- Pores > 0.91 nm: ω exhibits a mixed distribution (~50% at 0° and 90°), with the first layer retaining partial order due to surface interactions and outer layers showing increased rotational freedom.

Notably, ω behavior is consistent across surface types, confirming its dependence on pore size-induced steric effects rather than surface chemical properties.

#### 3.2.4. Dihedral Angle (τ): Molecular Chain Tortion

The dihedral angle (τ), defined by adjacent planes of four consecutive carbon atoms, remains stable at ~0° across all pore sizes, indicating that the n-octane chain maintains a rigid zigzag configuration. This rigidity persists despite pore size variations or layering, as van der Waals repulsion in confined spaces limits carbon-carbon bond rotation, consistent with the structural properties of short-chain alkanes. Notably, bulk and confined n-alkanes are known to retain non-zero gauche populations; therefore $\tau \approx 0^{\circ }$ at the wall should be read as reduced average deviation (trans-biased), not a fully rigid zigzag. This interpretation aligns with literature reports of incomplete all-trans adoption even under confinement [23].

### 3.3. Correlation Mechanism of Pore Size, Configuration, and Adsorption Performance

Pore size governs n-octane molecular behavior through the interplay of steric hindrance and potential field superposition, with the four angular parameters providing a quantitative description:

- Pores < 4.26 nm: Strong steric confinement and overlapping surface potentials impose orientational order: the tilt (θ) and azimuth (φ) show narrow, unimodal distributions (chains align in a fixed in-plane direction), and the chain torsion (*τ*) is locked into a single dominant conformation (predominantly trans), resulting in high adsorption density.
- Pores > 4.26 nm: Reduced steric and potential field effects lead to disordered configurations (dispersed θ and φ, mixed state of ω), with increased layering but stable density per layer.

This correlation elucidates how pore confinement modulates molecular organization and adsorption characteristics, critical for shale oil recovery.

## 4. Discussion

The four-angle parameter system (θ, φ, ω, and τ) overcomes the limitations of traditional qualitative descriptions by comprehensively quantifying molecular inclination, projection, plane rotation, and chain conformation. This approach establishes a quantitative link between molecular configurations and adsorption density, enabling a robust microscopic-macroscopic connection. Its versatility extends to other chain molecules (e.g., long-chain alkanes, fatty acids) and surfaces (e.g., montmorillonite, graphene) by simply adjusting the reference framework (e.g., lattice orientation). This method delivers a standardized, reusable tool for analyzing interfacial interactions, advancing the quantitative studies of configuration-performance relationships in shale oil and broader interface science.

The observed dichotomy—strongly ordered adsorption in sub-nanometer slits versus layered yet in-plane-broadened adsorption in ≥4–5 nm slits—is governed by two dimensionless controls: (i) the confinement ratio H/σ (pore width relative to molecular size) and (ii) the corrugation parameter $\kappa =\Delta U/k_{B}T$ that compares surface-field corrugation to thermal energy. Because H/σ is geometric and ΔU is set by the wall potential, moderate changes in temperature or pressure primarily rescale thermal broadening and interlayer packing but do not invert the ordering regimes identified at 323 K and 1 bar. Consistent with this, block-wise re-sampling of the present trajectories shows that the rank order of orientational metrics (narrow > wide in alignment; wide > narrow in layer multiplicity) remains unchanged within uncertainties. We therefore regard the trends reported here as qualitatively robust to moderate T–p variations, while deferring quantitative re-parameterization to future work.

## 5. Conclusions

This study introduces a novel four-angle parameter system—elevation angle (θ), azimuth angle (φ), rotation angle (ω), and dihedral angle (τ)—applied to molecular dynamics simulations to systematically quantitatively characterize the adsorption behavior of n-octane in kaolinite slit-like pores ranging from 0.45 to 14.05 nm. The results elucidate the intricate interplay between pore size, molecular configuration, and adsorption performance in shale systems.

Firstly, the four-angle parameters effectively capture the precise multi-dimensional response of n-octane in confinement: θ quantifies chain inclination relative to the kaolinite surface, φ reveals surface lattice-driven orientation, ω describes plane rotation influenced by steric hindrance, and τ characterizes internal torsional conformations, confirming the presence of a rigid extended alkane conformation. Together, these parameters quantify subtle pore-size–dependent responses of n-octane at the alkane–clay interface, beyond what qualitative descriptions can resolve.

Secondly, pore size governs molecular configurations and macroscopic properties through dual effects. Pore size governs the behavior via steric confinement (<0.55 nm) and surface-potential superposition (≤4.26 nm): narrow pores yield highly ordered, high-density monolayers, whereas beyond ~4.26 nm layering increases while in-plane order and per-layer density decline.

Thirdly, surface heterogeneity, particularly contrasting siloxane and aluminol surfaces, further modulates φ distributions. Ordered siloxane molecules align at φ at 60° and 120°, while dynamic hydroxides of the aluminol surfaces promote disorder, reflected in φ values. This is particularly evident by the appearance of double adsorption peaks at pore sizes > 0.91 nm, highlighting surface chemistry effects on orientation.

Finally, this quantification method overcomes the limitations of qualitative approaches, providing a standardized, transferable framework for analyzing alkane-clay interactions. The approach generalizes to other long-chain molecules and 2D materials, providing a standardized framework for cross-scale interfacial analysis.

## Data Availability

The original contributions presented in this study are included in the article. Further inquiries can be directed to the corresponding author(s).
